# Efficacy and safety of dosage-escalation of low-dosage esaxerenone added to a RAS inhibitor in hypertensive patients with type 2 diabetes and albuminuria: a single-arm, open-label study

**DOI:** 10.1038/s41440-019-0270-2

**Published:** 2019-06-25

**Authors:** Hiroshi Itoh, Sadayoshi Ito, Hiromi Rakugi, Yasuyuki Okuda, Satoshi Nishioka

**Affiliations:** 1 Department of Endocrinology, Metabolism and Nephrology; Keio University, School of Medicine, 35 Shinanomachi, Shinjuku, Tokyo, 160-8582 Japan; 2 Division of Nephrology, Endocrinology and Vascular Medicine; Department of Medicine, Tohoku University School of Medicine, 2-1 Seiryo-machi, Aoba, Sendai, Miyagi 980-8575 Japan; 3 Department of Geriatric and General Medicine, Osaka University Graduate School of Medicine, 2-2 Yamadaoka, Suita, Osaka 565-0871 Japan; 4 Daiichi Sankyo Co., Ltd., 1-2-58 Hiromachi, Shinagawa, Tokyo, 140-8710 Japan

**Keywords:** Albuminuria, Esaxerenone, Hypertension, Mineralocorticoid receptor blocker, Type 2 diabetes

## Abstract

The stimulation of mineralocorticoid receptors is linked to the development of hypertension and cardiovascular or renal damage in patients with diabetes, and the blockade of these receptors may be an effective treatment option. This open-label study with a 12-week treatment period assessed the antihypertensive (primary) and antialbuminuric (secondary) efficacy and safety of esaxerenone as an add-on therapy to a renin–angiotensin system inhibitor in hypertensive patients with type 2 diabetes and albuminuria (urinary albumin-creatinine ratio 30 to <1000 mg/g•Cr). Esaxerenone was administered over 12 weeks at a starting dosage of 1.25 mg/day, which was gradually titrated to 2.5 mg/day and 5 mg/day at weeks 4, 6, or 8 according to the dosage-escalation criteria based on serum K^+^ levels, the estimated glomerular filtration rate, and the likelihood/occurrence of hypotension. Of the 51 patients enrolled, 44 (86.3%) reached an esaxerenone dosage of 2.5 or 5 mg/day. The changes from the baseline in sitting systolic and diastolic blood pressures were −13.7 mmHg (*p* < 0.05) and −6.2 mmHg (*p* < 0.05), respectively. Significant decreases in blood pressure occurred regardless of age, baseline systolic blood pressure, glycated hemoglobin level, and estimated glomerular filtration rate. The urinary albumin-creatinine ratio decreased by 32.4% from the baseline (*p* < 0.05). Two consecutive serum K^+^ measurements ≥ 5.5 mEq/L occurred in one patient but resolved after dosage reduction. Esaxerenone showed antihypertensive and antialbuminuric effects and a low risk of hyperkalemia with dosage titration from 1.25 mg in Japanese hypertensive patients with type 2 diabetes and albuminuria receiving a renin–angiotensin system inhibitor.

## Introduction

Aldosterone, the final product of the renin–angiotensin system (RAS), is involved in the regulation of blood electrolytes and body fluid volume by acting on the mineralocorticoid receptor (MR), a nuclear receptor in renal tubular and intestinal epithelial cells, to promote Na^+^ reabsorption and K^+^ excretion [1]. Aldosterone also reduces the production of nitric oxide, a vascular relaxing factor, via MRs in vascular endothelial cells [2] and acts directly on vascular smooth muscle to constrict blood vessels [3, 4]. MR blockers, including spironolactone and eplerenone, have been developed and are commercially available as antihypertensive agents. Moreover, MR stimulation is associated not only with hypertension but also with organ damage. The sustained activation of renal MRs is implicated in metabolic diseases, such as diabetes, via RAS-dependent and independent mechanisms, and can ultimately cause kidney damage independent of blood pressure (BP) [5–7].

Hypertension frequently presents with type 2 diabetes as a comorbidity [8], and hypertensive patients with diabetes tend to be resistant to treatment [9–11]. Furthermore, patients with resistant hypertension have a high risk of cardiovascular events, so strict BP control is required [8]. For the treatment of hypertensive patients with diabetes and albuminuria, an angiotensin II receptor blocker (ARB) or an angiotensin converting enzyme (ACE) inhibitor is recommended as a first-line treatment [8, 12]. However, because a considerable proportion of hypertensive patients with diabetes cannot achieve the target BP or adequate end-organ protection with ARB or ACE inhibitors alone [13], add-on antihypertensive medications are required. Moreover, for those with albuminuria, antihypertensive medications with a renoprotective effect are required.

For patients with diabetic nephropathy, a significant reduction in BP has been achieved with the administration of an MR blocker as an add-on therapy to an ARB or ACE inhibitor [14–16]. However, one currently available MR blocker, eplerenone, is rarely used in hypertensive patients with renal dysfunction or type 2 diabetes. This is because, in Japan, eplerenone is contraindicated in hypertensive patients with diabetes and concomitant albuminuria, microalbuminuria, or proteinuria, or in patients with a creatinine clearance of <50 mL/min, due to the considerable risk of increased serum K^+^ levels seen in clinical studies [17–20].

Another MR blocker, spironolactone, can be used in these patient populations, but treatment is associated with sex hormone-related adverse drug reactions such as gynecomastia because of the low selectivity of spironolactone for MRs, and this is regarded as a clinically relevant problem [21, 22].

Esaxerenone (CS-3150) is a novel oral, nonsteroidal MR blocker that is highly selective for the MR, meaning that sex hormone-related adverse events should be less likely and that esaxerenone should have greater potency than spironolactone and eplerenone [23]. A series of phase 1 clinical studies demonstrated that esaxerenone is well tolerated and dose-dependently increases the plasma renin activity (PRA) and plasma aldosterone concentration (PAC) via MR blockade [24]. In a phase 2 study in patients with essential hypertension, a significant decrease in sitting BP was demonstrated after 12 weeks of esaxerenone treatment at daily doses of 2.5 mg and 5 mg [25]. In another study, esaxerenone treatment was associated with a reduction in the urine albumin-to-creatinine ratio (UACR) in patients with type 2 diabetes and albuminuria (NCT02345057, unpublished data). However, these data were not sufficient to support the antihypertensive and albuminuria-lowering effects of esaxerenone or to assess its influence on serum K^+^ levels in hypertensive patients with type 2 diabetes and albuminuria.

Therefore, this study primarily evaluated the antihypertensive efficacy and secondarily investigated the albuminuria-reducing effect of esaxerenone as an add-on therapy to a RAS inhibitor in hypertensive patients with type 2 diabetes and albuminuria. The safety of esaxerenone was also assessed in these patients.

## Methods

### Study design

**Fig. 1:**
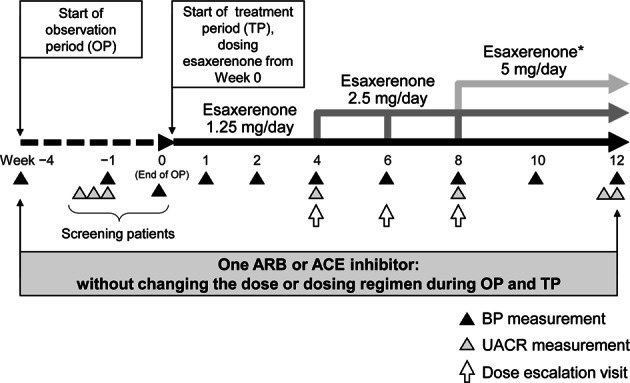
Study design. At week −1 of the observation period, the first morning void urine sample was collected for three consecutive days. At week 12 of the treatment period, the first morning void urine sample was collected for two consecutive days. The dosage escalation criteria are detailed in the Methods section. *Only patients who had their dosage escalated to 2.5 mg/day at week 4 were eligible for further escalation. *ARB* angiotensin receptor blocker, *ACE* angiotensin-converting enzyme

This multicenter, single-arm, open-label, dose-escalation study was conducted at 12 clinics in Japan from July 2016 to March 2017 (Fig. 1) over 16 weeks (a 4-week observation period and a 12-week treatment period). Patients started esaxerenone at an initial dosage of 1.25 mg/day after breakfast. The dosage was escalated to 2.5 mg/day at week 4, week 6, or week 8 of the 12-week treatment period. A subsequent dosage escalation to 5 mg/day esaxerenone occurred only at week 8 in patients who escalated to 2.5 mg/day esaxerenone at week 4 (Fig. 1). The increased dosages, 2.5 mg and 5 mg/day, were determined based on the results of a previous phase 2 dosage-escalation study [25].

To reduce safety risks in patients with contraindications for eplerenone, the following dosing regimen was employed: start at a low dosage (1.25 mg/day) followed by gradual escalation to 2.5 mg and then 5 mg/day according to the patient’s condition. All of the following dosage-escalation criteria were required to be met at week 4, week 6, and week 8 of the 12-week treatment period: a serum K^+^ level of <4.8 mEq/L; no decrease of ≥ 30% in the estimated glomerular filtration rate (eGFR) at the previous visit compared with week −1 in the observation period; and no observation of impending hypotension.

The dosage reduction criteria included a serum K^+^ level ≥ 6.0 mEq/L, two consecutive serum K^+^ levels ≥ 5.5–<6.0 mEq/L or severe hypotension during treatment with esaxerenone 2.5 mg or 5 mg/day. Treatment was discontinued if any of these criteria were met during treatment with esaxerenone 1.25 mg/day.

The study protocol was reviewed and approved by the institutional review board at each center and was conducted in accordance with the International Conference on Harmonization Guidelines for Good Clinical Practices and the ethical principles of the Declaration of Helsinki. All patients provided written informed consent.

### Patients

The included patients were aged 20–80 years; had a trough sitting systolic BP (SBP) of 140–<180 mmHg, a diastolic BP (DBP) of 80–<110 mmHg, a UACR 30–<1000 (mg/g•Cr), an eGFR ≥ 30 mL/min/1.73 m^2^ in the observation period; and had received treatment with a stable dosage and regimen of one ARB or ACE inhibitor during the 4-week observation period. Patients with secondary hypertension or hypertensive emergency, type 1 diabetes, or a serum K^+^ level < 3.5 mEq/L or ≥4.8 mEq/L were excluded.

### Prior and concomitant medications

The concomitant use of antihypertensive agents (ARBs, ACE inhibitors, calcium antagonists, or α/β-blockers), except for existing therapy with one ARB or ACE inhibitor, was prohibited during both the 4-week observation period and the 12-week treatment period. The use of glycyrrhiza, glycyrrhizin preparations, and nonsteroidal anti-inflammatory analgesics for more than five consecutive days was prohibited. Adrenocorticosteroids, immunosuppressants, K^+^ supplements, and ion exchange resins were also prohibited.

### Measurement of BP, UACR, and laboratory tests

The protocol for the BP measurements at each visit is described in a separate manuscript [25]. In brief, after 5 min of rest, the clinic sitting BP (HEM-7080IC; OMRON COLIN) was measured three times at each time point, and the mean of the three readings at each visit was used for the analyses. The baseline BP was the mean of readings taken at two visits: week −1 and 0 of the observation period. During esaxerenone treatment, the trough BP (24 h after the previous dose) was measured at weeks 1, 2, 4, 6, 8, 10, and 12 of the treatment period (Fig. 1).

Urine samples for the measurement of the UACR were collected at week –1 of the observation period and weeks 4, 8, and 12 of the treatment period. During the observation period, the first morning void urine sample was collected for three consecutive days before the day of the visit; if the values met the criteria (30–<1000 mg/g•Cr) at two or more time points, the mean of the latter two values was used as the baseline UACR. At the end of the study, at week 12 of the treatment period, the first morning void urine sample was collected for two consecutive days before the day of the visit, and the mean of the values was used as the final UACR. Urine samples were refrigerated by the patient from the time of collection until the study visit (Fig. 1).

All laboratory test parameters were measured by a central laboratory. K^+^ and creatinine (eGFR) were measured at weeks −1, 1, 2, 4, 6, 8, 10 and 12; other laboratory test parameters were measured at weeks −1, 4, 8 and 12. When testing showed a serum K^+^ level ≥ 5.5 mEq/L, a retest was performed immediately (within 3 days whenever possible).

### Efficacy endpoints

The primary endpoints were changes in trough sitting SBP and DBP from the baseline to the end of treatment. The end-of-treatment value used in the primary analysis was the mean of the values at weeks 10 and 12 of the treatment period. The last observation carried forward method was used to impute missing BP values.

The secondary endpoints were the changes over time in the trough sitting BP (SBP and DBP) and the percent change in the UACR from the baseline to the end of treatment. PAC and PRA were measured to assess the magnitude of MR inhibition by esaxerenone. An exploratory analysis was performed to determine the mean change and percent change over time in urinary markers of nephropathy, including 8-hydroxydeoxyguanosine (8-OHdG), angiotensinogen (AGT), β2-microglobulin (β2-MG), liver-type fatty acid binding protein (L-FABP), and N-acetyl-β-(D)-glucosaminidase (NAG).

### Safety endpoints

The safety endpoints included adverse events (AEs) and the incidence of increased serum K^+^ levels. This included the percentage of patients with a serum K^+^ level ≥ 5.5 mEq/L or ≥6.0 mEq/L on a single measurement or ≥5.5 mEq/L on two consecutive measurements. Laboratory test parameters were also evaluated; these included hematology, blood biochemistry, and urinalysis.

### Statistical analysis

The efficacy analysis was conducted in the full analysis set (FAS), which included patients who provided informed consent, met the inclusion criteria, took the study drug at least once, and had at least one efficacy measurement recorded. The safety analysis set (SAS) included patients who provided informed consent and were administered the study drug at least once.

The change in sitting BP (95% confidence interval [CI]) from the baseline to the end of treatment was calculated and compared using paired *t*-tests. Subgroup analyses of the antihypertensive effect of esaxerenone were performed by age, baseline SBP, glycated hemoglobin (HbA1c) level, eGFR at the baseline, and the dosage of esaxerenone at the end of treatment. The geometric mean percent changes and 95% CIs in UACR, PAC, PRA, and other urinary markers from the baseline to the end of treatment (week 12) were calculated, and paired *t*-tests were used to assess any differences using log-transformed values. Safety variables were summarized using descriptive statistics such as the mean, standard deviation (SD), and 95% CI. *Post hoc* analyses consisted of the following: the change in UACR stratified by the dosage of esaxerenone at the end of treatment and the statistical significance of the changes in BP, eGFR and serum K^+^ levels over time. These were assessed with paired *t*-tests. All reported p-values are two-sided and were not adjusted for multiple testing; *p*-values < 0.05 were considered statistically significant. The statistical analyses were performed with SAS System Release 9.3 (SAS Institute Inc., Cary, NC).

## Results

### Patient demographics

Of the 106 patients screened, 51 were enrolled, and 47 (92.2%) completed the study treatment. Four patients discontinued treatment: one withdrew consent, two discontinued as a result of AEs (thrombotic cerebral infarction and generalized rash), and one patient discontinued due to the physician’s advice.

The baseline patient characteristics are shown in Table 1. The mean age was 63.0 years, the mean HbA1c level was 6.8%, and the mean serum K^+^ level was 4.2 mEq/L. The mean sitting SBP/DBP at baseline was 158.7/89.0 mmHg, and 45.1% of the patients had an SBP ≥ 160 mmHg. The proportions of patients with an eGFR < 60 mL/min/1.73 m^2^ and a UACR ≥ 300 mg/g•Cr were 29.4 and 21.6%, respectively, while diabetic retinopathy and diabetic neuropathy were present in 52.9 and 33.3% of the patients, respectively.

**Table 1:** Baseline characteristics

	Esaxerenone (*n* = 51)
Male, *n* (%)	39 (76.5)
Age, years	63.0 ± 9.8
≥65 years, *n* (%)	24 (47.1)
Body mass index, kg/m^2^	26.3 ± 3.8
≥25 kg/m^2^, *n* (%)	33 (64.7)
Systolic BP, mmHg	158.7 ± 10.9
≥160 mmHg, *n* (%)	23 (45.1)
Diastolic BP, mmHg	89.0 ± 5.9
≥100 mmHg, *n* (%)	1 (2.0)
Diabetic complications, *n* (%)	34 (66.7)
Diabetic retinopathy	27 (52.9)
Diabetic neuropathy	17 (33.3)
Dyslipidemia, *n* (%)	39 (76.5)
Hyperuricemia, *n* (%)	13 (25.5)
Serum K^+^, mEq/L	4.2 ± 0.3
≥4.5 mEq/L, *n* (%)	11 (21.6)
eGFR, mL/min/1.73 m^2^	73.1 ± 19.5
<60 mL/min/1.73 m^2^, *n* (%)	15 (29.4)
HbA1c, %	6.8 ± 0.6
<6.9%, *n* (%)	29 (56.9)
≥6.9–<7.4%, *n* (%)	13 (25.5)
≥7.4%, *n* (%)	9 (17.6)
Fasting plasma glucose, mg/dL	127.0 ± 23.8
UACR, mg/g•Cr	
Median (range)	97.1 (32.3–967.1)
Geometric mean (95% CI)	123.0 (92.4, 163.6)
≥300 mg/g•Cr, *n* (%)	11 (21.6)
Basal antihypertensive agents, *n* (%)	
ARB	45 (88.2)
ACE inhibitor	6 (11.8)
Antihyperglycemic agents, *n* (%)	48 (94.1)
DPP4 inhibitor	29 (56.9)
SGLT2 inhibitor	6 (11.8)
GLP-1 receptor agonist	5 (9.8)
Others	45 (88.2)
HMG-CoA reductase inhibitor, *n* (%)	25 (49.0)

Values are mean ± standard deviation or number of patients (%), unless otherwise specified

*ACE* angiotensin converting enzyme, *ARB* angiotensin II receptor blocker, *BP* blood pressure, *DPP4* dipeptidyl peptidase-4, *eGFR* estimated glomerular filtration rate, *GLP-1* glucagon-like peptide-1, *HbA1c* glycated hemoglobin, *HMG-CoA* hydroxymethylglutaryl-CoA, *SGLT2* sodium-glucose co-transporter-2, *UACR* urine albumin-to-creatine ratio

The maximum dosage of esaxerenone was 2.5 mg/day in 25 patients (49.0%) and 5 mg/day in 19 patients (37.3%), based on the prespecified dosage titration method.

### Efficacy

**Fig. 2:**
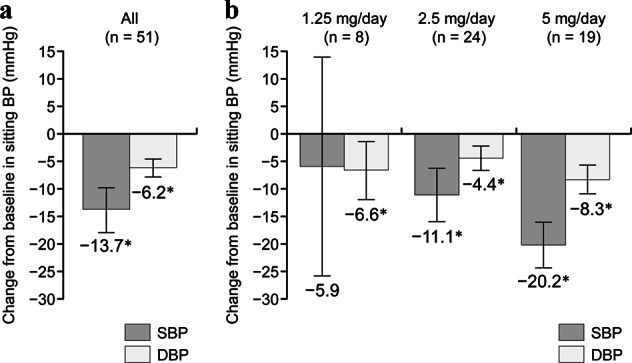
Changes in sitting blood pressure (BP) from the baseline to the end of treatment in all patients (**a**) and stratified by the end-of-treatment dosage of esaxerenone (**b**). Data are presented as the mean difference and 95% confidence intervals. *p < 0.05, paired *t*-test for changes from the baseline. The last observation carried forward method was used. Full analysis set, *n* *=* 51. *DBP* diastolic BP, *SBP* systolic BP

**Fig. 3:**
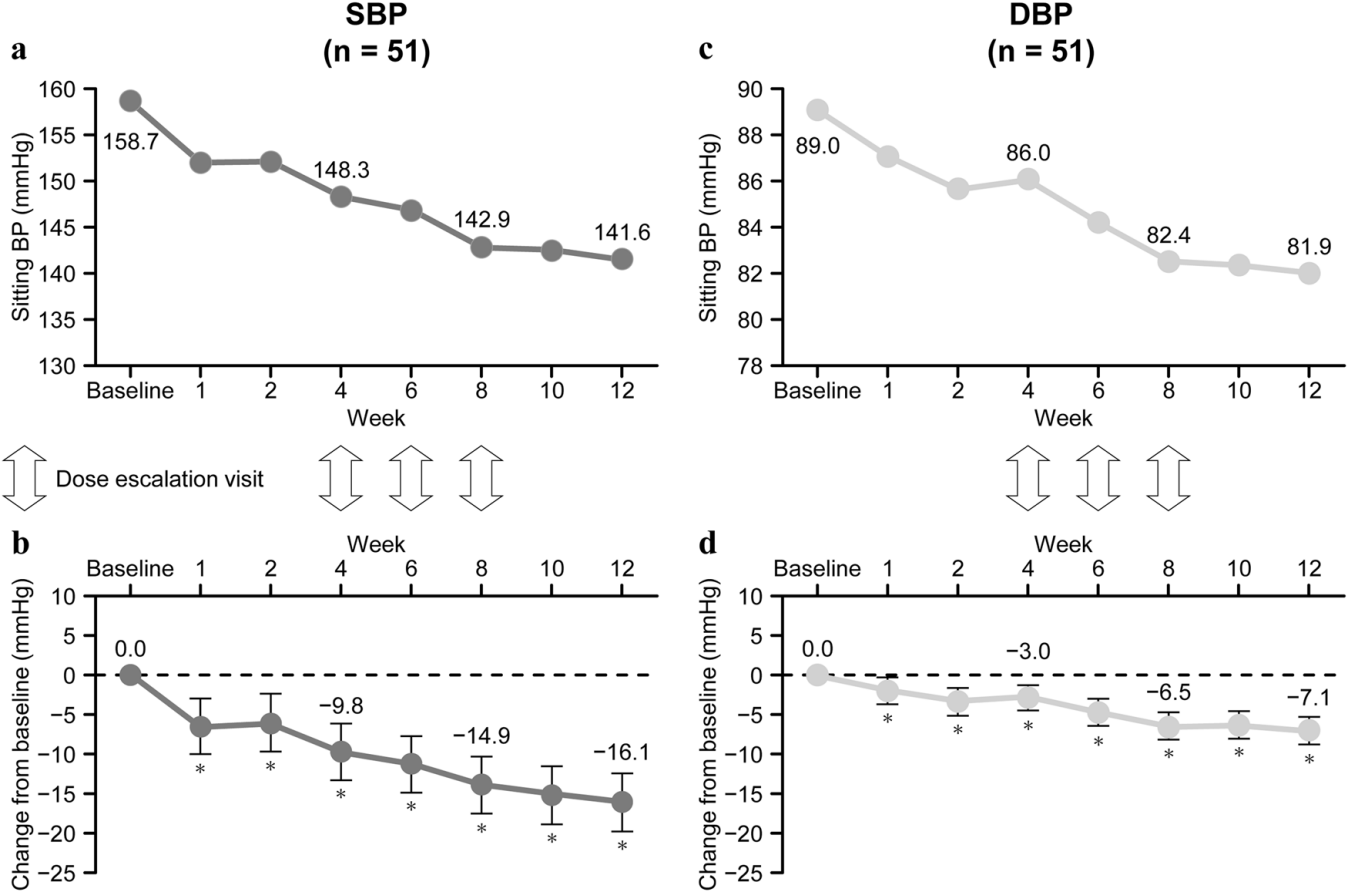
Changes in sitting blood pressure (BP) over time during treatment with esaxerenone. Sitting SBP (**a**), change from the baseline in SBP (**b**), sitting DBP (**c**) and change from the baseline in DBP (**d**). Data are means and 95% confidence intervals. *p < 0.05, paired *t*-test for changes from baseline (*post hoc* analysis). Dosage escalations occurred at weeks 4, 6, and 8. Full analysis set, *n* *=* 51. *DBP* diastolic BP, *SBP* systolic BP

There were significant reductions in the mean (95% CI) sitting trough SBP and DBP from the baseline to the end of treatment: −13.7 (−17.6, −9.8) mmHg (*p* < 0.05) and −6.2 (−7.8, −4.6) mmHg (*p* < 0.05), respectively (Fig. 2). Reductions from the baseline in both SBP and DBP were observed in patients who were receiving esaxerenone 2.5 or 5 mg/day at the end of the treatment period (−11.1/−4.4 mmHg and −20.2/−8.3 mmHg, respectively; both *p* < 0.05) (Fig. 2). No significant reductions in SBP were observed in patients taking esaxerenone 1.25 mg/day. Sitting SBP/DBP decreased incrementally during the treatment period, especially after each esaxerenone dosage titration visit (*p* < 0.05) (Fig. 3). The significant antihypertensive effects of esaxerenone were consistent across all patient subgroups in the subgroup analysis (by sex, age <65 vs. ≥ 65 years, baseline SBP <160 vs. ≥160 mmHg, baseline HbA1c <6.9 vs. ≥ 6.9%, and baseline eGFR <60 vs. ≥ 60 mL/min/1.73 m^2^) (all *p* < 0.05 for the reduction in BP vs. the baseline) (Supplementary Figure 1).

**Fig. 4:**
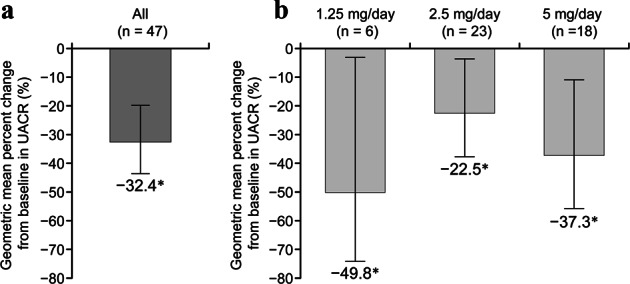
Geometric mean percent changes in the urine albumin-to-creatine ratio (UACR) from the baseline to the end of treatment in all patients (**a**) and stratified by the end-of-treatment dosage of esaxerenone (**b**). Data are geometric mean and 95% confidence interval. *p < 0.05, paired *t*-test for changes from the baseline (**b** *post hoc* analysis). Full analysis set, *n* *=* 47

There was a significant −32.4% reduction in the UACR from the baseline to week 12 (95% CI −43.4, −19.2; *p* < 0.05) (Fig. 4). In a *post hoc* subgroup analysis based on the end-of-treatment dosage of esaxerenone, there was a ≥20% significant reduction in UACR in all dosage groups (Fig. 4). Other *post hoc* analyses investigating changes in the UACR from the baseline in patient subgroups showed the following reductions: −32.9% versus −30.3% in patients with baseline UACR < 300 mg/g Cr (*n* = 38) versus ≥ 300 mg/g Cr (*n* = 9); −32.3% versus −32.4% in patients with baseline eGFR < 60 mL/min/1.73 m^2^ (n = 12) versus ≥ 60 mL/min/1.73 m^2^ (*n* = 35); and −27.2% versus −46.9% in males (*n* = 36) versus females (*n* = 11). None of the differences between subgroups were statistically significant.

The baseline geometric mean (95% CI) values for PAC and PRA were 80.9 (69.1, 94.7) pg/mL and 1.08 (0.71, 1.64) ng/mL/h, respectively. PAC and PRA were both significantly increased at week 12 by 40.2% (95% CI 21.5, 61.8) and 123.9% (95% CI 57.9, 217.5), respectively (Supplementary Table 1). Of the urinary markers assessed, only β2-MG was significantly decreased from the baseline (Supplementary Table 1).

### Safety

The incidence of AEs was 49.0% (25/51), and the most common AEs were viral upper respiratory tract infection (19.6%) and increased serum K^+^ level (11.8%) (Table 2). The incidences of total AEs were comparable between male and female patients (48.7% and 50.0%, respectively). Treatment with esaxerenone was generally well tolerated, and the majority of AEs were mild. Two patients (3.9%) discontinued treatment due to AEs. One of these AEs was thrombotic cerebral infarction, which was determined to be related to the study drug by an investigator because it occurred 1 week after the initiation of esaxerenone treatment. The patient developed right-sided upper limb weakness and fell. Cerebral infarction was diagnosed using magnetic resonance imaging, and the patient was admitted to the hospital for treatment. The paralysis resolved 16 days after the event. Because the event required hospitalization, it was regarded as a serious adverse event (SAE). The other AE leading to treatment discontinuation was systemic rash. This was judged by the investigator to be unrelated to the study drug (but related to other concomitant agents).

**Table 2:** Summary of safety events

	Esaxerenone (*n* = 51)
At least one AE, *n* (%)	25 (49.0)
TEAEs reported in ≥3% of patients, n (%)	
Viral upper respiratory tract infection	10 (19.6)
Increased serum K^+^	6 (11.8)
Back pain	2 (3.9)
At least one drug-related AE, *n* (%)	4 (7.8)
Thrombotic cerebral infarction	1 (2.0)
Increased serum K^+^	3 (5.9)
Treatment discontinued due to an AE, *n* (%)	2 (3.9)
Thrombotic cerebral infarction	1 (2.0)
Rash generalized	1 (2.0)
Discontinuation due to increased serum K^+^	0 (0)
Dose reduction due to serum K^+^ ≥5.5 to <6.0 mEq/L on two consecutive measurements, *n* (%)	1 (2.0)

*AE* adverse event, *TEAE* treatment-emergent adverse event

Four patients reported at least one drug-related treatment-emergent AE. In addition to the above patient with thrombotic cerebral infarction, increased serum K^+^ levels occurred in three patients (mild for all patients). There were no sex hormone-related AEs.

The mean serum K^+^ level significantly increased by 0.25 mEq/L at week 1 from 4.20 mEq/L at the baseline. Subsequently, the serum K^+^ levels remained stable, and the maximum mean change from the baseline was 0.45 mEq/L at week 10 (Supplementary Figure 2). Two patients (3.9%) had serum K^+^ levels ≥ 5.5 mEq/L at any visit during the treatment period, but these levels did not exceed 6.0 mEq/L in either patient (Supplementary Table 2). One patient (2.0%), a 73-year-old female with a baseline serum K^+^ level of 4.2 mEq/L, had a serum K^+^ level ≥ 5.5 mEq/L on two consecutive measurements at week 5, but both were <6.0 mEq/L (5.5 and 5.9 mEq/L). After the esaxerenone dosage was reduced from 2.5 to 1.25 mg/day, her serum K^+^ levels decreased to 5.1 mEq/L, and this patient completed 12 weeks of study treatment without requiring any further intervention to control her K^+^ levels. No patients discontinued esaxerenone treatment due to increased serum K^+^ levels.

After the start of esaxerenone treatment, the eGFR decreased significantly from the baseline but returned to baseline levels by 1 week after the end of treatment (Supplementary Figure 3).

## Discussion

This study evaluated the antihypertensive effect, the albuminuria-lowering effect, and safety of esaxerenone in hypertensive patients with type 2 diabetes and albuminuria concomitantly receiving an ARB or ACE inhibitor. Significant reductions in SBP and DBP (−13.7 and −6.2, *p* < 0.05 vs baseline) were observed during 12 weeks of treatment with esaxerenone, and the antihypertensive effects of the regimen studied (dosage escalation from 1.25 mg/day to 5 mg/day) were similar to those observed in studies of patients with essential hypertension treated with esaxerenone 2.5 mg/day [25] (NCT02890173, unpublished data). In the analysis stratified by final esaxerenone dosage, dosage-dependent, significant reductions in SBP and DBP were observed with dosages of 2.5 mg/day and 5 mg/day esaxerenone (−11.1 and −4.4, and −20.2 and −8.3, respectively; both *p* < 0.05). There was no significant reduction in SBP with esaxerenone 1.25 mg/day, but the number of patients treated with this dosage was small, and the variation was large. Although the number of patients in this study was relatively small (*n* = 51), consistent antihypertensive effects were observed with esaxerenone, regardless of the baseline patient characteristics such as sex, age, SBP, HbA1c level, and eGFR.

Several similar studies have evaluated the antihypertensive effect of adding an MR blocker to a RAS inhibitor in type 2 diabetic patients with albuminuria. In a study of 30 randomized patients, Saklayen et al. reported that the mean SBP decreased from 153.64 ± 25.95 to 141.60 ± 16.54 mmHg (*p* *=* 0.01) in patients receiving spironolactone 100 mg/day in addition to an ARB or ACE inhibitor [26]. However, in contrast to our study, the DBP was not significantly reduced. In another study, the DBP/SBP was reduced by a mean (95% CI) of 7 (2, 12)/3 (1–6) mmHg with spironolactone 25–50 mg/day (*n* = 29) vs a placebo (*n* = 30) in patients with type 2 diabetes and macroalbuminuria receiving long-term treatment with an ACE inhibitor or ARB [27]. Rossing et al. reported that the addition of spironolactone to an existing ACE inhibitor or ARB antihypertensive therapy reduced SBP/DBP by 10/5 mmHg versus a placebo in a study of 21 patients with diabetic nephropathy [28]. Another study investigated the antihypertensive effects of eplerenone 50 or 100 mg/day added to ACE inhibitor therapy in patients with type 2 diabetes (*n* = 91 and *n* = 86, respectively) and reported significant reductions in BP from the baseline [15]. In contrast to our study, the eplerenone study allowed the addition of amlodipine 2.5–10 mg/day from week 4 onwards if needed to achieve the target BP (≤130/80 mmHg), and add-on amlodipine was required in 70% and 58% of patients in the eplerenone 50 mg/day and 100 mg/day groups, respectively [15]. Taken together, the available data and current findings suggest that esaxerenone is likely to have antihypertensive effects that are at least comparable to those of spironolactone and eplerenone.

These findings also indicate that esaxerenone has clinically significant antihypertensive effects, resulting in strict BP control in hypertensive patients with type 2 diabetes who may be resistant to antihypertensive treatment. This supports the use of esaxerenone as an add-on therapy for patients with treatment-resistant hypertension with comorbid diabetes, including MR-associated hypertension, which has two subtypes. One subtype includes patients with elevated plasma aldosterone levels, such as those associated with primary aldosteronism, and those who experience “aldosterone escape” or “aldosterone breakthrough” during therapy with ACE inhibitors or ARBs [29]. The other subtype includes patients with obesity, diabetes mellitus or chronic kidney disease (CKD) and normal plasma aldosterone levels [29]. The factors that are believed to play a role in the pathogenesis of MR-associated hypertension with normal plasma aldosterone levels include MR activation by pathways other than high aldosterone levels (such as increased MR levels), increased MR sensitivity, and MR overstimulation by other factors [29, 30]. Indeed, the majority of patients in this study had MR-associated hypertension with normal plasma aldosterone concentrations.

Albuminuria is reported to be a strong prognostic factor [31–33]. A reduction in the UACR in patients with diabetic nephropathy is correlated with a reduction in the occurrence of adverse renal events (end-stage renal disease). In the current study, we observed not only antihypertensive effects but also a significant reduction in the UACR during esaxerenone treatment, which was consistent with the results of another study of esaxerenone (NCT02345057, unpublished data). In the end-of-treatment dosage analysis, the UACR reduction was −49.8%, even when the final esaxerenone dosage was 1.25 mg/day. Although the number of patients was small (*n* = 6), this suggests that esaxerenone dosages even lower than 2.5 mg/day might reduce the UACR in some sensitive patients. The ability of MR blockers to reduce proteinuria has previously been established in CKD patients [14, 15, 28], and the results of the present study also showed that esaxerenone treatment reduced albuminuria when added to an ARB or ACE inhibitor. Some studies have reported that MR activation induces renal tubular injury through inflammation and fibrosis [34, 35]. Esaxerenone has been shown to inhibit the progression of renal dysfunction in an experimental rat model of renal dysfunction [23, 36]. In this clinical study, the only renal dysfunction biomarker that significantly decreased during treatment with esaxerenone was β2-MG. Nevertheless, esaxerenone is expected to have a renoprotective effect via the suppression of renal tubular injury, especially when used for a longer treatment duration than that in our study. This suggests that esaxerenone may be an appropriate treatment option for preventing the progression of diabetic nephropathy. Two phase 3 studies of esaxerenone in this important patient population are ongoing (JapicCTI-173695 and JapicCTI-173696).

Hyperkalemia is a known dosage-dependent side effect of MR blockers such as spironolactone and eplerenone [37–40]. The addition of an MR blocker to an ACE inhibitor or ARB significantly increases the risk of hyperkalemia (relative risk 3.74, 95% CI 2.30–6.09, *p* < 0.00001) [41]. Clinically significant hyperkalemia (e.g., serum K^+^ ≥ 6.0 mEq/L) can have serious consequences, including impaired cardiac rhythm [42, 43]. In this study, no patients had a serum K^+^ level ≥ 6.0 mEq/L. Only one patient had serum K^+^ ≥ 5.5 mEq/L on two consecutive measurements, but a reduction in esaxerenone dosage lowered the level to <5.5 mEq/L, and the patient was able to complete the study. The proportion of patients (3.9%) with serum K^+^ levels ≥ 5.5 mEq/L in this study was similar to that in studies of esaxerenone in patients with essential hypertension (NCT02890173 and NCT02722265, unpublished data). This incidence of hyperkalemia was also comparable to that in a previous study of low-dosage spironolactone therapy (25 mg/day; less than the dosage indicated for treating hypertension) added to a RAS inhibitor in diabetic patients with albuminuria [28, 44, 45]. Although the serum K^+^ level significantly increased from week 1 in our study, the extent of the change was similar to that with low-dosage spironolactone [28, 44, 45]. For eplerenone, the incidence of hyperkalemia during clinical studies in hypertensive patients with type 2 diabetes and albuminuria was high (serum K^+^ > 5.5 mEq/L in 32.3% of patients treated with eplerenone 200 mg alone and 37.9% in those receiving eplerenone 200 mg plus enalapril 10 mg), leading to eplerenone being contraindicated in this patient group [46]. Subsequent clinical study data showed that the incidence of hyperkalemia decreased after eplerenone dosage reduction [14]. However, the prescription of eplerenone for hypertensive patients with renal dysfunction or diabetes with albuminuria is still contraindicated [46].

Hyperkalemia is also a common AE associated with ARBs and ACE inhibitors [47–49]. However, in this study, when esaxerenone was added to an existing ARB or ACE inhibitor therapeutic regimen with a careful dosage escalation/reduction protocol, no patients had to discontinue treatment due to increased serum K^+^ levels, and one patient with an increased serum K^+^ level was able to continue treatment with esaxerenone after a dosage reduction. These findings indicate that hyperkalemia due to the addition of esaxerenone to therapy with an ARB or ACE inhibitor can be considered clinically manageable by adjusting the dosage from 1.25 to 5 mg/day. By escalating the dosage according to each patient’s serum K^+^ level, renal function and BP, esaxerenone demonstrated acceptable safety in this patient population, who were also receiving an ARB or an ACE inhibitor. However, monitoring serum K^+^ levels at an appropriate frequency in the clinical setting would still be required.

The reductions in the eGFR observed in this study were not considered to constitute a clinically relevant safety concern because they were small in magnitude, only occurred immediately after the initiation or dosage escalation of esaxerenone, and returned to the baseline levels after the end of treatment. This is consistent with previous reports showing a slight and transient reduction in the eGFR after initiating therapy with an MR blocker or RAS inhibitor [50, 51]. The decrease in the eGFR appeared to be associated with hemodynamic changes because it corresponded with alterations in BP. Overall, no new safety concerns with esaxerenone were identified in this study, and all AEs were consistent with those observed in other studies of esaxerenone [25] (NCT02345057, NCT02890173, and NCT02722265, unpublished data).

This study has several limitations, including a small number of patients, a single ethnicity population (Japanese), and the lack of a comparator. These should be taken into account when interpreting and extrapolating our findings.

## Conclusions

The results of this study show that treatment with esaxerenone administered via a gradual stepwise titration approach from 1.25 to 2.5 and 5 mg/day achieves additional antihypertensive efficacy while reducing albuminuria when added to an ARB or ACE inhibitor in Japanese hypertensive patients with type 2 diabetes and albuminuria, and the safety profile of the regimen was manageable. In particular, potential increases in serum K^+^ levels can be minimized by starting treatment at a lower dosage (1.25 mg/day) and then increasing up to 5 mg/day.

## Supplementary information

Supplementary table 1

Supplementary table 2

Supplementary figure 1

Supplementary figure 2

Supplementary figure 3
