# Supplementary material for: Efficacy and safety of dosage-escalation of low-dosage esaxerenone added to a RAS inhibitor in hypertensive patients with type 2 diabetes and albuminuria: a single-arm, open-label study
Source: Hypertens Res. 2019 Jun 25;42(10):1572–81. doi: 10.1038/s41440-019-0270-2 (PMC8075891; doi:10.1038/s41440-019-0270-2)
Supplement: Supplementary file 1 — Supplementary table 1 [file 41440_2019_270_MOESM1_ESM.docx]

## Supplementary table 1 Changes in PAC, PRA, and urinary markers of nephropathy

**PAC and PRA**

|  | **Baseline** | **Week 12** | **Percent change** |
| --- | --- | --- | --- |
| PAC, pg/mL | 80.9 (69.1, 94.7)  (n = 36) | 110.0 (93.1, 129.9)  (n = 32) | 40.2%*  (21.5, 61.8) |
| PRA, ng/mL/h | 1.08 (0.71, 1.64)  (n = 34) | 2.38 (1.63, 3.47)  (n = 30) | 123.9%*  (57.9, 217.5) |

**Urinary markers of nephropathy**

|  | **Baseline** | **Week 12** | **Percent change** |
| --- | --- | --- | --- |
| 8-OHdG, ng/mL•Cr | 10.01 (9.04, 11.08)  (n = 47) | 9.72 (8.88, 10.64)  (n = 41) | −1.7%  (−11.0, 8.5) |
| AGT, ng/mL•Cr | 0.236 (0.184, 0.302)  (n = 49) | 0.189 (0.139, 0.257)  (n = 46) | −16.0%  (−32.1, 3.9) |
| β2-MG, µg/L•Cr | 2.177 (1.533, 3.092)  (n = 49) | 1.303 (0.899, 1.888)  (n = 46) | −37.3%*  (−54.6, −13.2) |
| L-FABP, µg/g•Cr | 3.690 (2.890, 4.712)  (n = 50) | 3.378 (2.604, 4.383)  (n = 45) | −5.4%  (−22.4, 15.3) |
| NAG, IU/L•Cr | 0.045 (0.038, 0.053)  (n = 49) | 0.045 (0.038, 0.054)  (n = 47) | 7.4%  (−5.7, 22.2) |

Values are geometric mean (95% CI), unless otherwise specified. 8-OHdG, 8-hydroxydeoxyguanosine; β2-MG, β2-microglobulin; AGT, angiotensinogen; CI, confidence interval; L-FABP, liver-type fatty acid binding protein; NAG, N-acetyl-β-(D)-glucosaminidase; PAC, plasma aldosterone concentration; PRA, plasma renin activity

*p<0.05
