# Supplementary material for: Efficacy and safety of dosage-escalation of low-dosage esaxerenone added to a RAS inhibitor in hypertensive patients with type 2 diabetes and albuminuria: a single-arm, open-label study
Source: Hypertens Res. 2019 Jun 25;42(10):1572–81. doi: 10.1038/s41440-019-0270-2 (PMC8075891; doi:10.1038/s41440-019-0270-2)
Supplement: Supplementary file 2 — Supplementary table 2 [file 41440_2019_270_MOESM2_ESM.docx]

## Supplementary table 2 Incidence of hyperkalemia during the treatment period

|  | **Esaxerenone (n = 51)** |
| --- | --- |
| Serum K^+^ ≥5.5 mEq/L, n (%) | 2 (3.9) |
| Serum K^+^ ≥6.0 mEq/L, n (%) | 0 (0.0) |
| Serum K^+^ ≥5.5 mEq/L on two consecutive measurements, n (%)^1^ | 1 (2.0)^1^ |

^1^In this patient, serum K^+^ was <6.0 mEq/L on both measurements and decreased to <5.5 mEq/L after the esaxerenone dosage was reduced from 2.5 to 1.25 mg/day; the patient subsequently completed the 12-week treatment period.
