# Supplementary figures and images for: Efficacy and safety of dosage-escalation of low-dosage esaxerenone added to a RAS inhibitor in hypertensive patients with type 2 diabetes and albuminuria: a single-arm, open-label study
Source: Hypertens Res. 2019 Jun 25;42(10):1572–81. doi: 10.1038/s41440-019-0270-2 (PMC8075891; doi:10.1038/s41440-019-0270-2)

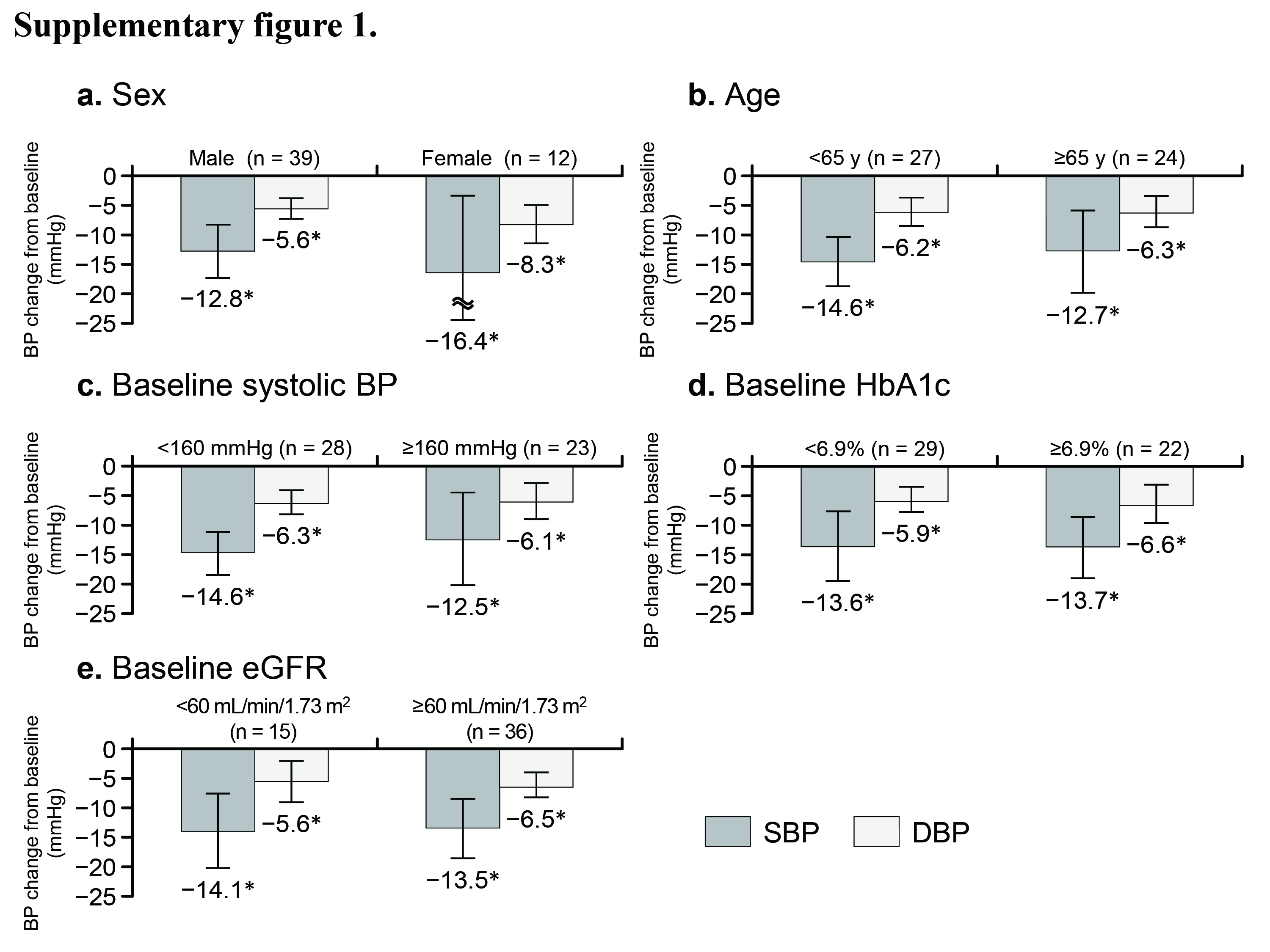

Supplement: Supplementary file 3 — Supplementary figure 1 [file 41440_2019_270_MOESM3_ESM.tif]

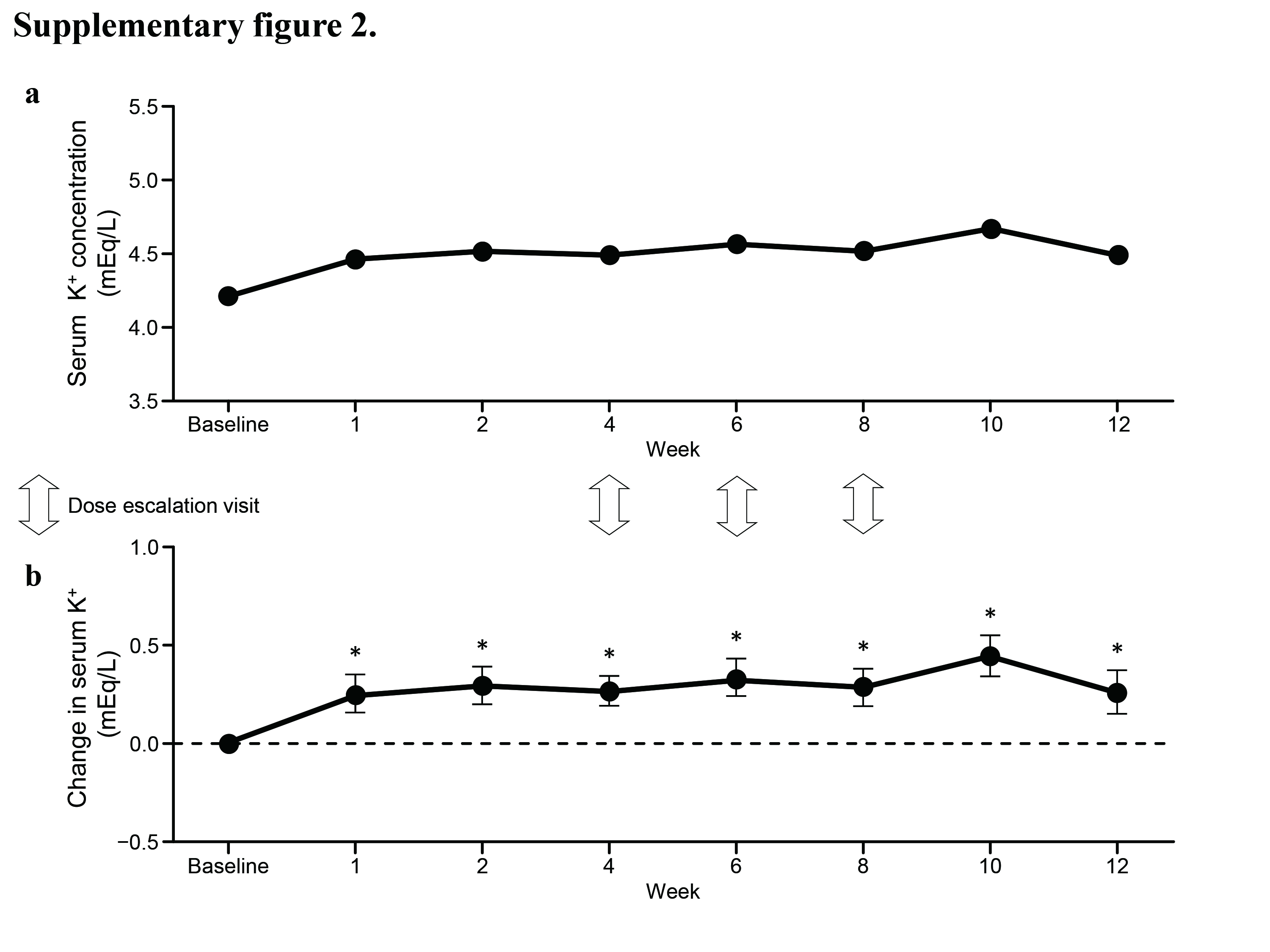

Supplement: Supplementary file 4 — Supplementary figure 2 [file 41440_2019_270_MOESM4_ESM.tif]

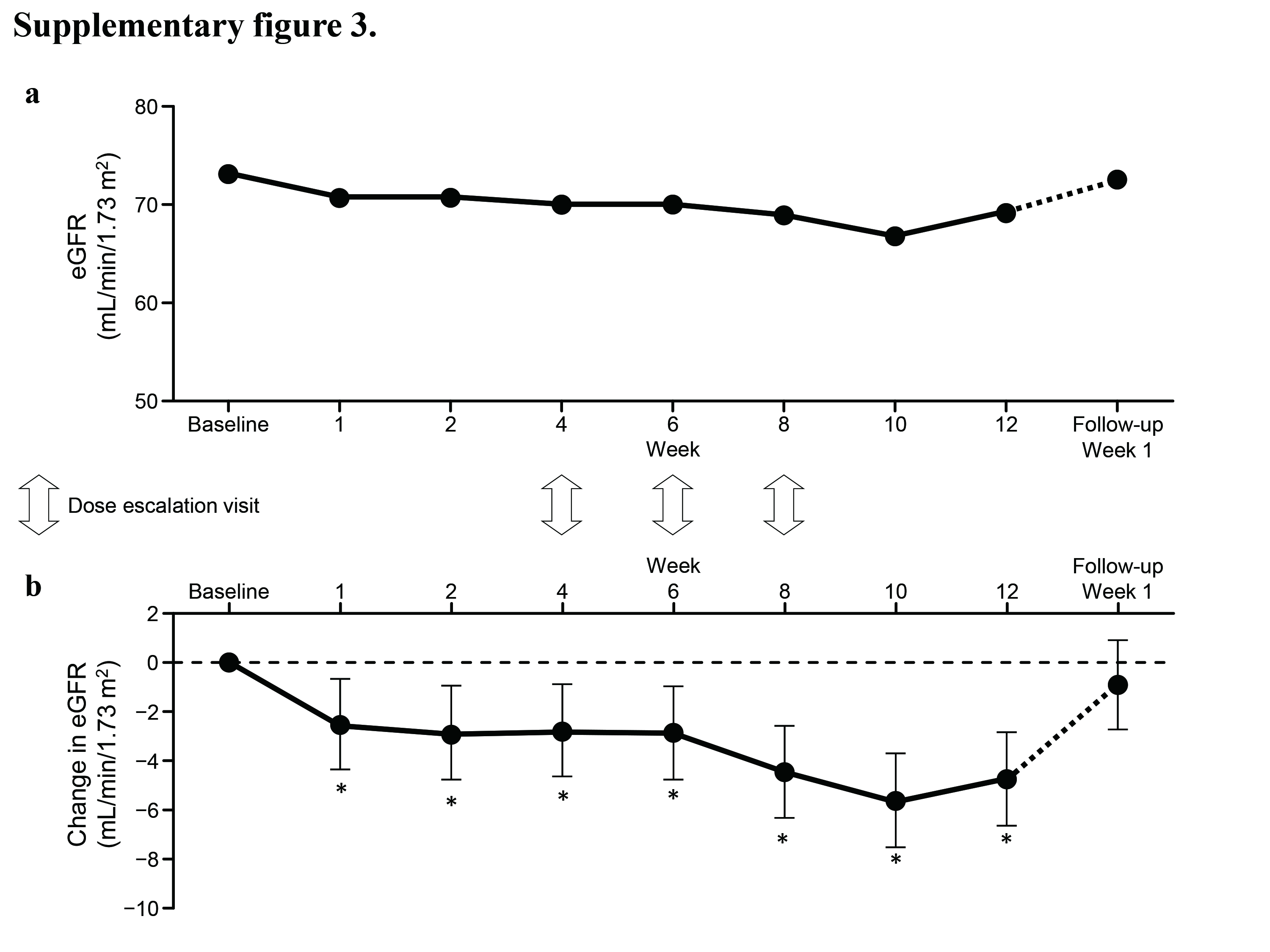

Supplement: Supplementary file 5 — Supplementary figure 3 [file 41440_2019_270_MOESM5_ESM.tif]
